# Flipping the switch

**DOI:** 10.7554/eLife.31082

**Published:** 2017-09-19

**Authors:** Xavier Pierrat, Alexandre Persat

**Affiliations:** Division of Life SciencesGlobal Health Institute and Institute of Bioengineering, EPFLLausanneSwitzerland

**Keywords:** Vibrio cholerae, biofilm, matrix protein, protease, RbmA, VPS, Other

## Abstract

A structural switch controls the architecture of *Vibrio cholerae* biofilms by mediating the interactions between two matrix components.

**Related research article** Fong JC, Rogers A, Michael AK, Parsley NC, Cornell WC, Lin YC, Singh PK, Hartmann R, Drescher K, Vinogradov E, Dietrich LE, Partch CL, Yildiz FH. 2017. Structural dynamics of RbmA governs plasticity of *Vibrio cholerae* biofilms. *eLife* **6**:e26163. doi: 10.7554/eLife.26163

As single-celled organisms bacteria are exposed to a variety of stresses, but their ability to form multicellular structures called biofilms helps them to grow and survive in challenging environments. In addition to offering protection against predators, antimicrobial treatments and other forms of stress, being in a biofilm also makes bacteria more effective in spreading diseases (Costerton et al., 1995).

The cells in a biofilm are embedded in an elastic matrix made of polysaccharides, DNA and proteins that have been secreted by the bacteria, and, like bricks in a wall, they are precisely organized (Drescher et al., 2016). However, in spite of its toughness, the matrix must remain permeable to nutrients and flexible so that the biofilm can continue to grow.

To achieve this, bacteria use a set of matrix proteins that are patterned in space and time (Berk et al., 2012). However, it is still unclear how these matrix proteins interact to simultaneously remain a connected and flexible multicellular structure. Now, in eLife, Carrie Partch, Fitnat Yildiz and co-workers – including Jiunn Fong of the University of California, Santa Cruz as first author – report new insights into how the architecture of the matrix of the bacterium *Vibrio cholerae* is regulated (Fong et al., 2017).

Despite improved and ongoing vaccination programs, *V. cholerae* still causes major pandemics of cholera in high-risk areas such as Yemen, where over half a million people have recently been infected, and thousands have been killed (World Health Organization, 2017a; World Health Organization, 2017b). The bacterium spreads rapidly through contaminated water sources and intestinal infections – a cycle that is promoted by the formation of biofilms.

Because the lifecycle of *V. cholerae* depends on switching between planktonic and biofilm states, the components of the matrix must remain dynamic. Two of the most important components are a protein called RbmA and a polysaccharide called VPS (Figure 1A; Teschler et al., 2015). Each component performs a specific role, which is partly determined by its position during the formation of the biofilm. RbmA helps cells to stick together and to form clusters, while VPS is needed to develop a three-dimensional cellular structure. In the matrix, RbmA can exist as a monomer or two RbmA proteins can combine to form a dimer (Figure 1B,C; Giglio et al., 2013). However, until now it was not known how these different states influence the architecture of the biofilm, or how RbmA forms dimers in the first place.

**Figure 1.**
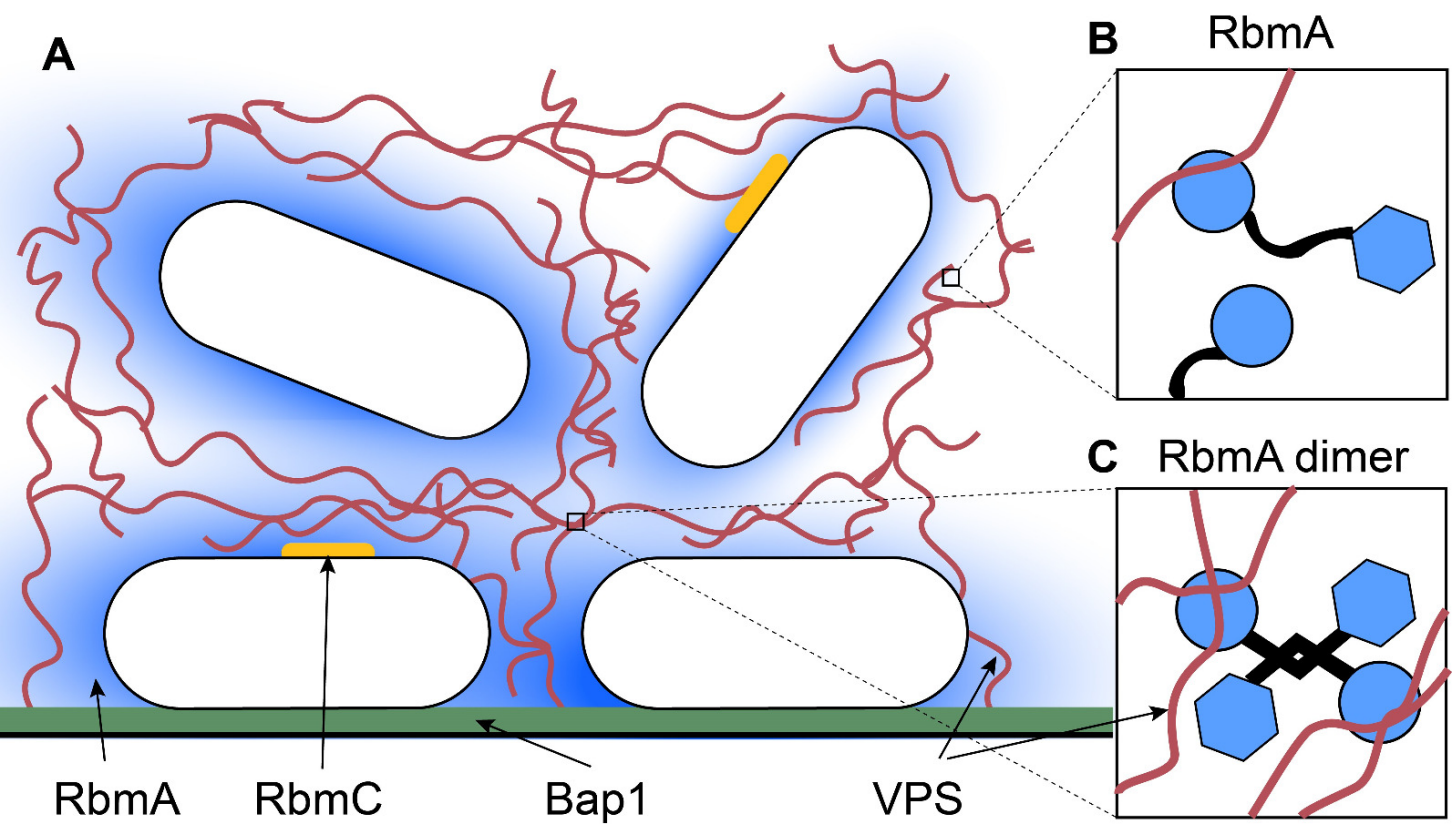
Inside a biofilm. (**A**) *Vibrio cholera*e (white) secretes four main matrix proteins: RbmA (blue), RbmC (orange), Bap1 (green) and VPS (red strands). (**B**) An RbmA monomer (blue and black) binds to VPS (red). (**C**) An RbmA dimer can bind to several strands of VPS to create a stable mesh across the matrix.

Fong et al. demonstrated that far from being inert, the matrix components are dynamic structures that interact with each other to maintain the biofilm architecture. Using a technique called nuclear magnetic resonance spectroscopy, Fong et al. found that the dimers can adopt one of two conformations – an ordered loop or a disordered loop. However, only the ordered-loop conformation can enable RbmA to form the antiparallel dimers, which are less prone to degradation than the disordered-loop monomers.

Fong et al. further discovered that both the monomers and the dimers have the ability to bind to VPS. However, RbmA dimers cross-link VPS to form a more solid complex that is anchored into the matrix. When Fong et al. genetically modified RbmA to only form dimers, the proteins concentrated in the center of the matrix instead of building a stable mesh across it. This affected the mechanical properties of the matrix, and the biofilms appeared smooth with a lower cell density. However, removing RbmA led to visually similar biofilms, despite the absence of the cross-linked protein complexes.

These findings raise a plethora of questions: for example, what causes the changes in the viscous properties of the matrix – the RbmA dimer or VPS? And does the position of RbmA in the matrix cause the architectural defects in mutant biofilms? It remains unclear what triggers the formation of the RbmA dimer. One possibility may be that the increasing internal forces generated within a growing biofilm could toggle the conformational change, in a manner that is similar to other bacterial adhesion proteins (Persat et al., 2015). If this was indeed the case, the biofilm matrix could be considered as a ‘smart hydrogel’ that can modulate adhesion depending on its mechanical environment.

The study by Fong et al. builds a foundation for future studies exploring the role of further matrix components of *V. cholerae* and other organisms. The principles of biofilm formation might not be specific to the microbial world – other living systems also depend on the dynamic properties of their extracellular matrices. In humans, for example, the architecture and dynamics of the extracellular matrix secreted by embryonic cells tightly control how the tissue and organs develop (Rozario and DeSimone, 2010). It has also been shown that force-induced dynamic interactions between extracellular matrix components play a central role in wound healing (Kubow et al., 2015). There is clearly much that we can learn by digging deeper into the matrix.
